# Aptitude visuelle à la conduite automobile: exemple des candidats au permis de conduire à Libreville

**DOI:** 10.11604/pamj.2015.22.147.6811

**Published:** 2015-10-16

**Authors:** Hassane Souhail, Prudence Assoumou, Hilda Birinda, Emmanuel Mve Mengome

**Affiliations:** 1 Service Ophtalmologie, Hôpital d'Instruction des Armées Omar Bongo Ondimba, Libreville, Gabon

**Keywords:** Aptitude visuelle, permis de conduire, acuité visuelle, vision des couleurs, Libreville, Visual aptitude, driving license, visual acuity, color vision, Libreville

## Abstract

L'objectif était d’évaluer l'aptitude visuelle à la conduite automobile des candidats au permis de conduire à Libreville. Il s'agissait d'une étude transversale, descriptive et analytique, qui s'est déroulée à Libreville pendant la période du 4 avril 2012 au 14 juillet 2012 (soit 4 mois et 10 jours). La population d’étude concernait les candidats soumis aux épreuves d'obtention du permis de conduire. Nous avons inclus dans notre travail, les candidats, ayant donné leur consentement par écrit et exclus ceux refusant d'adhérer à l'enquête. Les variables étudiées concernaient l’âge, le sexe, la population d’étude, l'activité professionnelle, l'acuité visuelle de loin et de près, la vision des couleurs, la catégorie du permis de conduire, et l'aptitude visuelle à la conduite automobile. La saisie et l'analyse des données ont été collectées au moyen d'une fiche d'enquête standardisée; après vérification et validation, elles ont été saisies sur le logiciel Excel Windows et analysées sur le logiciel Epi Info version 3.5.1. L’âge moyen des 406 candidats était de 29 ans ± 6,65 ans avec des extrêmes allant de 17 ans à 52 ans. Les hommes représentaient 283 (69,7%) et les femmes 123 (30,3%), soit un ratio de 2,3. Les fonctionnaires étaient retrouvés dans 39,4 % des cas, suivi des élèves-étudiants dans 33,5%. Dans notre population d’étude, 71 sur 406 candidats avaient une baisse de l'acuité visuelle de loin, soit 17,5%. Dans notre série, nous avons retrouvés 34 candidats âgés de 40 ans et plus, et seulement 14 candidats (41,2%) avaient une baisse de l'acuité visuelle de près. La quasi-totalité des patients avaient une vision de couleurs normale (99,5%), cependant 2 candidats avaient une vision de couleurs anormale, soit une prévalence de 0,5%. Dans notre échantillon, 403 (99,3%) sollicitaient un permis de conduire de catégorie léger (perms A, A1, B, F) et 3 (0,7%) sollicitaient un permis de conduire de type lourd ( C,D,E). Les candidats pour la catégorie de permis légers étaient inaptes dans 10 cas soit 2,5% de la population d’étude. Toutefois, tous les candidats à la catégorie de permis lourd étaient aptes à la conduite automobile. Nos résultats montraient que 2,5% des candidats étaient inaptes à la conduite automobile et que l'examen ophtalmologique n'avait pas été réalisé pour la quasi- totalité des candidats dans le cadre de l'obtention du permis de conduire. Rappelons toutefois que, l’évaluation de l'aptitude visuelle du futur conducteur constitue un acte de prévention de sécurité routière et qu'il revient donc à l'ophtalmologiste de délivrer le certificat d'aptitude visuelle à la conduite, pour s'assurer que le candidat au permis de conduire ne présente pas d'affection visuelle incompatible avec la conduite automobile.

## Introduction

De nos jours, conduire une automobile est un acte ordinaire, courant voire insignifiant mais toutefois, il n'en demeure pas sans danger, car est susceptible d'entraîner pour soi-même ainsi que pour les autres, un risque d'accident. Cependant, la fonction visuelle reste indispensable à la conduite automobile car, les informations nécessaires au conducteur lui sont fournies par l’œil dans presque la quasi-totalité des cas, soit 90% [1, 2]. Aussi, on estime que 20% des responsables d'accident de la circulation ont un trouble visuel [3]. Le rôle de la vue dans la survenu des accidents de la circulation a été bien documenté [3, 4, 5] De ce fait, la prévention des accidents de la circulation passe par l’évaluation de l'aptitude visuelle à la conduite automobile des candidats au permis de conduire. En Europe et dans le reste du monde, tout candidat au permis de conduire devra passer un examen ophtalmologique approprié, pour s'assurer qu'il ne présente pas d'affection visuelle incompatible avec la conduite automobile. Par ailleurs, l'application des normes minimales d'aptitude visuelle est d'actualité pour toutes les catégories de permis de conduire [6, 7]; une mesure qui a contribué à une évolution favorable et à l'amélioration des statistiques de sécurité routière [4, 8, 9]. En Afrique, des travaux ont été réalisés visant à atteindre cet objectif [9, 10]. Cependant, les accidents de la circulation y constituent un fardeau croissant et méritent des efforts de recherches proportionnels [11]. Au Gabon, on estime à près de 23000 accidents de la circulation par an [12]. L'incidence de la vision dans la survenue de ces accidents de la circulation n'a jamais été étudiée. Etant donné qu'un conducteur porteur d'anomalies visuelles constitue un risque pour la sécurité routière [13], il nous a paru important d’élaborer des stratégies pour une conduite automobile sécuritaire. Afin donc de participer à la mise en place des politiques d’évaluation de la vision chez les candidats au permis de conduire, nous avons initié ce travail dont le but est d’évaluer l'aptitude visuelle à la conduite automobile des candidats au permis de conduire à Libreville.

## Méthodes

Il s'agissait d'une étude transversale, descriptive et analytique, qui s'est déroulée du 4 Avril 2012 au 14 Juillet 2012 (soit 4 mois et 10 jours) au Centre national d'examen du permis de conduire, avec l'accord de la Direction Générale des transports terrestres à Libreville, capitale administrative et politique du Gabon. La population de l’étude concernait l'ensemble des candidats au permis de conduire de Libreville, rencontrés après les épreuves théoriques ou pratiques organisées par le Centre national d'examen du permis de conduire, les mercredi et samedi de la semaine pendant la période de l’étude. Nous avons inclus dans notre travail, les candidats soumis aux épreuves d'obtention du permis de conduire, ayant donné leur consentement par écrit et avons exclus, les candidats refusant d'adhérer à l'enquête. Les variables étudiées concernaient l’âge, le sexe, la population d’étude, l'activité professionnelle, l'acuité visuelle de loin et de près, la vision des couleurs, la catégorie du permis de conduire sollicitée, aptitude visuelle au permis de conduire. La saisie et l'analyse des données ont été collectées au moyen d'une fiche d'enquête standardisée. Après vérification et validation, elles ont été saisies sur le logiciel Excel windows et analysées sur le logiciel Epi Info version 3.5.1. La distribution des variables quantitatives a été décrite par les moyennes, écart-types et extrêmes. Par contre, les paramètres qualitatifs ont été décrits en termes d'effectifs et de pourcentages. Le test Khi-2 et le test exact de Fisher ont été utilisés pour la comparaison des proportions. Lorsque les effectifs étaient inférieurs à 5, les tests de correction de Yates et de Fisher ont été appliqués pour les variables qualitatives. Tous ces tests statistiques ont été réalisés au seuil de significativité inférieur à 5% (p < 0.05). Les données ont été recueillies à l'aide d'un questionnaire individuel standardisé, administré en mode face à face par l'enquêteur principal ayant suivi une formation au préalable sur les techniques d'examen. Les candidats ont été reçus individuellement dans une salle aménagée dans les locaux du Centre national d'examen du permis de conduire. Le questionnaire a été administré après consentement écrit de chaque candidat. Le remplissage du questionnaire était fait par l'enquêteur selon les réponses données par le candidat. L'examen ophtalmologique a consisté à mesurer l'acuité visuelle de loin et de près, puis à réaliser le test d'Ishihara. Les optotypes utilisés étaient des planches cartonnées. L'acuité visuelle de loin, a été étudiée à l'aide de l’échelle de Monoyer, placée à 5 mètres du sujet examiné. La vision a été testée d'abord en monoculaire puis en binoculaire. L'acuité a été mesurée sans et avec correction optique lorsque celle existait déjà. La vision normale correspondait à une acuité visuelle en vision de loin de 10/10 pour un œil, sans correction. Les candidats au permis déclarés aptes à la conduite automobile sont ceux qui ont répondus aux exigences suivantes:

### Pour les permis légers

Acuité visuelle binoculaire ≥ 5/10, obtenue éventuellement avec correction optique.

Acuité visuelle > 6/10 si monophtalmie.

### Pour les permis lourds

Acuité visuelle d'au moins 8/10 à l’œil le meilleur et d'au moins 5/10 à l’œil le moins bon, obtenue éventuellement avec correction optique.

L'acuité visuelle de près a été mesurée chez les candidats âgés de 40 ans et plus à l'aide du test de Parinaud, placé à 33 cm du sujet examiné. La vision a été testée d'abord en monoculaire puis en binoculaire. L'acuité a été mesurée sans et avec correction optique lorsque celle existait déjà. La vision normale correspondait à la lecture du niveau P2 sur le texte de Parinaud pour un œil sans correction optique. Les candidats ayant obtenus une acuité visuelle de loin inférieure à 10/10, une acuité visuelle de près au-dessus du niveau P2, sur au moins un œil et qui ne portaient pas de correction, ont été adressés à un ophtalmologiste de leur choix pour un examen ophtalmologique complet. La vision des couleurs, a été réalisée en binoculaire, en utilisant le test d'Ishihara. Tous les candidats dyschromates ont été informés de leur déficit et ont été adressés chez un ophtalmologiste pour meilleure investigation et prise en charge éventuelle. La vision des couleurs normale correspondait à l'identification correcte des tables pseudo-isochromatiques.

## Résultats

**Age**: l’âge moyen des 406 candidats au permis de conduire était de 29 ans ± 6,65 ans avec des extrêmes allant de 17 ans à 52 ans. Pour les besoins de l’étude, l’âge a été regroupé en 5 tranches. La tranche d’âge de (20-29) ans représentait plus de la moitié de la population étudiée soit 56,9%.

**Le sexe**: sur les 406 candidats au permis de conduire examinés, 283 étaient des hommes (69,7%) et 123 des femmes (30,3%) soit un ratio (H/F) de 2,3.

**L'activité professionnelle**: les fonctionnaires étaient retrouvés dans 39,4 % des cas, suivi des élèves-étudiants dans 33,5 % des cas.

### Aspects cliniques

**Les différents types de permis de conduire**: la quasi-totalité des candidats (97,8%) au permis de conduire avaient sollicité la catégorie B (Tableau 1).

**Tableau 1:** Les différents types de permis de conduire

Types de permis	Effectif	Pourcentage (%)
**Permis légers**		
Permis A	2	0,5
Permis A1	4	1,0
Permis B	**397**	**97,8**
Permis F	0	0,0
**Permis lourds**		
Permis C, D	3	0,7
Permis E	0	0,0
**TOTAL**	**406**	**100**

**L'acuité visuelle de loin**: dans notre population d’étude, 71 candidats sur 406 au total, soit 17,5% avaient une baisse de l'acuité visuelle de loin (Tableau 2).

**Tableau 2:** Répartition des candidats au permis de conduire selon la baisse de l'acuité visuelle de loin

BAV de loin	Effectif	Pourcentage (%)
Non	335	82,5
Oui	71	17,5
**Total**	**406**	**100,0**

**Baisse de l'acuité visuelle de loin et port de correction**: parmi les candidats ayant une baisse de l'acuité visuelle de loin, 52,1% portaient une correction (Tableau 3).

**Tableau 3:** Relation entre la baisse d'acuité visuelle de loin et port de correction chez les candidats au permis de conduire

Paramètres	BAV présentes N=71 (%)	BAV absentes N=335(%)	P
**Port de correction**			**0,000**
Oui	37 (63,8)	21 (36,2)	
Non	34 (9,8)	314 (90,2)	

**L'acuité visuelle de prés**: rappelons que la vision de près ne concernait que les candidats âgés de 40 ans et plus. Parmi nos 34 candidats âgés de 40 ans et plus, 14 candidats (41,2%) avaient une baisse de l'acuité visuelle de près (Tableau 4).

**Tableau 4:** Répartition des candidats au permis de conduire selon la baisse de l'acuité visuelle de près

BAV de près	Effectif	Pourcentage (%)
Non	30	58,8
Oui	**14**	**41,2**
**Total**	**34**	**100,0**

**Baisse de l'acuité visuelle de prés et port de correction**: parmi nos 14 candidats âgés de 40ans et plus, 6 soit 23,1% avaient une baisse de l'acuité visuelle de près et ne portaient pas de correction (Tableau 5).

**Tableau 5:** Relation entre la baisse d'acuité visuelle de près et port de correction chez les candidats au permis de conduire

Paramètres	BAV de prèsprésentes N=14 (%)	BAV de près absentes N=20(%)	P
**Port de correction**			**0,000**
Oui	8 (100,0)	0 (0,0)	
Non	6 (23,1)	20 (76,9)	

**La vision des couleurs**: la quasi-totalité des patients avaient une vision de couleurs normale (99,5%), cependant 2 candidats avaient une vision de couleurs anormale, soit une prévalence de 0,5 %.

**Aptitude des candidats à la conduite automobile**: dans notre échantillon, 403 (99,3%) sollicitaient un permis de conduire de catégorie léger (permis A, A_1_, B, F) et 3 (0,7%) sollicitaient un permis de conduire de type lourd (C, D, E).

**Aptitude des candidats à la catégorie de permis légers**: les candidats pour la catégorie de permis légers inaptes à la conduite automobile étaient un effectif de 10 soit 2,5% de la population d’étude (Tableau 6).

**Tableau 6:** Répartition des candidats selon la catégorie de permis léger et l'acuité visuelle binoculaire de loin avec ou sans correction

Paramètres	Permis légers N (%)
**Acuité visuelle binoculaire**	
≥ 5/10	393 (96,8)
< 5/10	10 (2,5)
**Total**	**403 (99,3)**

**Aptitude des candidats à la catégorie de permis lourds**: tous les candidats pour la catégorie de permis de permis lourd sont aptes à la conduite automobile (Tableau 7).

**Tableau 7:** Répartition des candidats selon la catégorie de permis lourd et l'acuité visuelle de loin avec ou sans correction

Paramètres	Permis lourds N=3 (%)
Acuité visuelle pour le meilleur œil	
**≥ 8/10**	**3 (0,7)**
**< 8/10**	0 (0,0)
Acuité visuelle l’œil le moins bon	
**≥ 5/10**	**3 (0,7)**
**< 5/10**	0 (0,0)

## Discussion

Notre étude est la première réalisée au Gabon et la seconde en Afrique subsaharienne après celle du Bénin en 1997 [14].

### Population de l’étude

Nous avons examiné 406 candidats au permis de conduire, un échantillon qui se rapproche de celui des travaux de Pointer au Royaume-Uni [15] et d Adekoya et al [14] au Nigéria. Cependant, sur quelques études faites en Afrique [16–19] et dont une faite en France [2], les groupes étudiées variaient entre 99 à 221 sujets.

### Age

Dans notre série, la tranche d’âge de ans représentait plus de la moitié de la population étudiée soit 56,9%. Ce résultat est similaire aux travaux d'Oussa et al [14] qui avaient rapporté 56,66% des cas pour cette même tranche d’âge à Cotonou.

### Sexe

Dans notre échantillon, les hommes représentaient 69,7% et les femmes 30,3%, soit un sex-ratio (H/F) de 2,3. Ce chiffre est loin derrière celui de Pointer au Royaume-Uni [15], où le sexe féminin représentait plus de la moitié de la population d’étude, soit 56%. Alors que pour la plupart des travaux menées en Afrique [16–19], les échantillons sont constitués uniquement d'hommes.

### Activité professionnelle

La majorité des candidats au permis de conduire était des fonctionnaires soit 39,4%, suivi des élèves-étudiants 33,5% des cas, du secteur informel dans 17,5% des cas et 9,6% étaient sans profession. Ceci pourrait s'expliquer par le fait que la voiture est un moyen de transport vulgarisé dans de nombreux pays [17], y compris au Gabon. Elle faciliterait l'exécution des tâches quotidiennes et fait donc partie intégrante de la notion de qualité de vie [17].

### Prévalence de la baisse de l'acuité visuelle

Il serait judicieux d'entrée de jeu de rapporter que dans notre échantillon aucun des candidats aux permis de conduire n'avait de monophtalmie. Dans notre série, 17,5% des candidats au permis de conduire âgés de 17 à 52 ans avaient une baisse de l'acuité visuelle de loin et 41,2% des candidats âgés de 40 à 52 ans avaient une baisse de l'acuité visuelle de près. Bien que nous n'ayons pas déterminé les étiologies de la baisse de l'acuité visuelle, les études antérieures montrent que les erreurs réfractives sont des étiologies fréquentes de la baisse de l'acuité visuelle chez les conducteurs. Ainsi, nos résultats sont proches d'une étude faite à Ibadan au Nigeria [19] avec une prévalence de 16,7%. Au Benin, cette proportion est de 13,0% [14].

### Baisse de l'acuité visuelle et âge

Dans notre série, il existait un lien significatif entre l’âge moyen et la survenue de la baisse de l'acuité visuelle de près chez les candidats, avec p =0,000. Ce résultat rejoint celui des travaux de Bekibele et al au Nigéria [19], dans lesquelles l'hypermétropie était liée à l’âge chez des conducteurs. Rappelons que l'hypermétropie est une erreur réfractive qui se traduit cliniquement par la baisse de l'acuité visuelle de près.

### Baisse de l'acuité visuelle et port de correction

Nous avons trouvé une relation entre le port de correction et la survenue de la baisse de l'acuité visuelle (de près et de loin), avec p =0,000. Cependant, parmi les candidats ayant une baisse de l'acuité visuelle de loin, moins de la moitié (47,9 %) ne portaient pas de correction. De même, pour les candidats âgés de 40 à 52 ans ayant une baisse de l'acuité visuelle de près, 23,1 % d'entre eux ne portaient pas de correction.

### Prévalence de la vision des couleurs anormale

Dans notre série, la prévalence des anomalies de la vision des couleurs était de 0,5%. Notre chiffre se rapproche de celui d'Omolase et al [10] au Nigéria qui n'avaient pas retrouvé d'anomalie de la vision des couleurs chez les conducteurs commerciaux de 22 ans et plus. Cependant, Adekoya et al [17] au Nigéria retrouvaient une prévalence des anomalies de la vision des couleurs de 4,3%. Par ailleurs, bien que nous ayons retrouvé des anomalies de la vision des couleurs chez quelques candidats au permis de conduire, celles-ci ne requièrent aucune exigence pour la conduite automobile [8].

### Baisse l'acuité visuelle de loin et de la vision des couleurs anormale

Il n'existait aucun lien significatif entre la baisse de l'acuité visuelle de loin et la vision des couleurs anormale, avec p >0,05.

### Aptitude des candidats à la conduite automobile

La notion d'inaptitude est définie pour un candidat atteint d'une affection visuelle et qui ne respecte pas aux normes minimales fixées par les autorités en charge de la délivrance du permis de conduire. Ainsi, dans notre échantillon, la proportion les candidats inaptes à la conduite automobile étaient 2,5%. Ce résultat se rapproche de celui retrouvé à Cotonou [14] avec 4,4% des sujets inaptes à la conduite automobile. Par ailleurs, d'autres études faites chez les conducteurs commerciaux ont trouvés des proportions supérieures à nos chiffres: 12,1% au Ghana [15], 11,5% à Ilorin au Nigéria [16] et 8,4% dans l’état d'Osun au Nigéria [20]. Cette disparité de résultats semble être liée à l'hétérogénéité des réglementations sur les normes minimales d'aptitude visuelle à la conduite automobile.

### Examen d'aptitude visuelle à la conduite automobile

Dans notre étude, la quasi-totalité (98,4%) des candidats n'avaient pas bénéficié d'un examen ophtalmologique pouvant juger de leur aptitude visuelle à la conduite automobile. Nos résultats sont proches de ceux d'Adekoya et al [17], Oladehinde et al [20] qui sont respectivement de 84,0% et 83,6 %. Les résultats d'Omolase et al [10] rejoignent ceux cités précédemment. Ceci se justifie par le fait que les pays en développement n'effectuent pas régulièrement des tests de dépistage pour la délivrance ou le renouvellement du permis de conduire, incitant les conducteurs à prendre leur vue pour acquis.

## Conclusion

Notre étude avait pour objectif général d’évaluer l'aptitude visuelle des candidats au permis de conduire à Libreville, par la mesure de l'acuité visuelle et la vision des couleurs. Ainsi, nos résultats montraient que 2,5% des candidats étaient inaptes à la conduite automobile. L’évaluation de l'aptitude du futur conducteur reposait sur des critères légaux conformément aux normes actuelles, notamment européennes. L'examen ophtalmologique n'avait pas été réalisé pour la quasi- totalité des candidats dans le cadre de l'obtention du permis de conduire. L’évaluation de l'aptitude visuelle du futur conducteur constitue un acte de prévention de sécurité routière. Il a pour objectif de diminuer le nombre et la gravité des accidents de la route. Un conducteur porteur d'anomalies visuelles peut ne pas percevoir un éventuel danger ou en prendre conscience tardivement pour réagir convenablement. La fonction visuelle demeure donc indispensable à la conduite automobile; Elle fait partie des critères qui déterminent l'aptitude à la conduite sur le plan médical. C'est à l'ophtalmologiste que revient le rôle de délivrer le certificat d'aptitude visuelle à la conduite.
